# Prevalence of Hepatitis B and Hepatitis C in Migrants from Sub-Saharan Africa Before Onward Dispersal Toward Europe

**DOI:** 10.1007/s10903-022-01448-z

**Published:** 2023-01-14

**Authors:** Frhat M. A. Saaed, Jerry E. Ongerth

**Affiliations:** 1 Department of Zoology, College of Arts and Sciences, Benghazi University, Al Kufra, Libya; 2 Environmental Engineering, University of Wollongong, Wollongong, NSW 2522 Australia

**Keywords:** hepatitis B, hepatitis C, Prevalence, Migrants, Sub-Saharan Africa, Al Kufra, Libya

## Abstract

Viral hepatitis is a global health care challenge due to its worldwide distribution, chronic persistence, complications, and high prevalence with unchecked conditions in areas like sub-Saharan Africa. A high proportion of asymptomatic infections allows serious complications and poses infection risk to destination populations. This study aimed to determine the prevalence of both HBV and HCV among 3248 migrants from different parts of sub-Saharan Africa newly arrived at Kufra, Libya, a remote agricultural North African city. All these migrants were required by the Libyan authorities to undergo a complete medical check-up for different purposes such as joining new jobs, and obtaining licenses for trade and commerce. UAT sera from 3248 migrants, aged 18–53 years, attending the Al Kufra city hospital from January 01 to December 31, 2019, were screened for HBsAg and anti-HCV antibody by rapid tests and positive samples were further tested by ELISA method. The results showed that 761/3248 (23.4%) of the migrants were positive for HBV and 1014/3248 (31.2%) were positive for HCV. Migrants from sub-Saharan Africa carry high rates of HBV and HCV infection. This suggests the importance of increased attention to actions to deal with findings among positive migrants, and for awareness about risks of transmission to the local population. Study results indicate the value of routine migrant monitoring, the need for awareness in destination country health authorities, and the potential for impact on migrant destination populations.

## Introduction

Hepatitis is a worldwide health problem with disproportionate geographic distribution features [1–4]. Infectious diseases accompany disadvantaged populations through migration patterns from underdeveloped and security threatened regions [5–7]. The blood- and bodily fluid-borne hepatitis infections, hepatitis B (HBV) and hepatitis C (HCV) are particular important [8–12]. National and international health agencies estimate worldwide nearly 300 million HBV infections accounting for nearly 800,000 deaths, and about 60 million HCV infections accounting for 300,000 deaths [1, 12]. The highest endemic HBV and HCV concentrations are in tropical and subtropical regions of Africa, Asia, and the Americas [6]. Combined with social and economic migration pressures from Africa and the Middle East, attention has been focused on the flow of infectious diseases in migrant populations [5, 7]. Libya, in its geographic position between sub-Saharan Africa and Europe, is a focal point for migrating populations from high endemic HBV and HBC regions to the South [13, 14]. This study sought to quantify the extent of these infections in current migrant populations at a major migration route transit point toward Europe.

Migrants and refugees from troubled African regions funnel through sub-Saharan areas and travel poorly established routes through trackless desert to reach the farthest Southeastern Libyan city, Al Kufra, and the road leading to the Mediterranean [6, 15]. Al Kufra city is the located in southeast Libya, about 1000 km to the nearest point on the Mediterranean coast, and 1700 km by road from Tripoli, the capital city and shortest route to Italian territory. Libya has international borders with Egypt in the east and with Sudan and Chad in the south. Migration routes to and through Libya are complex and vary to meet changing circumstances as described by the United Nations High Commission on Refugees (UNHCR) [15].

**Fig. 1:**
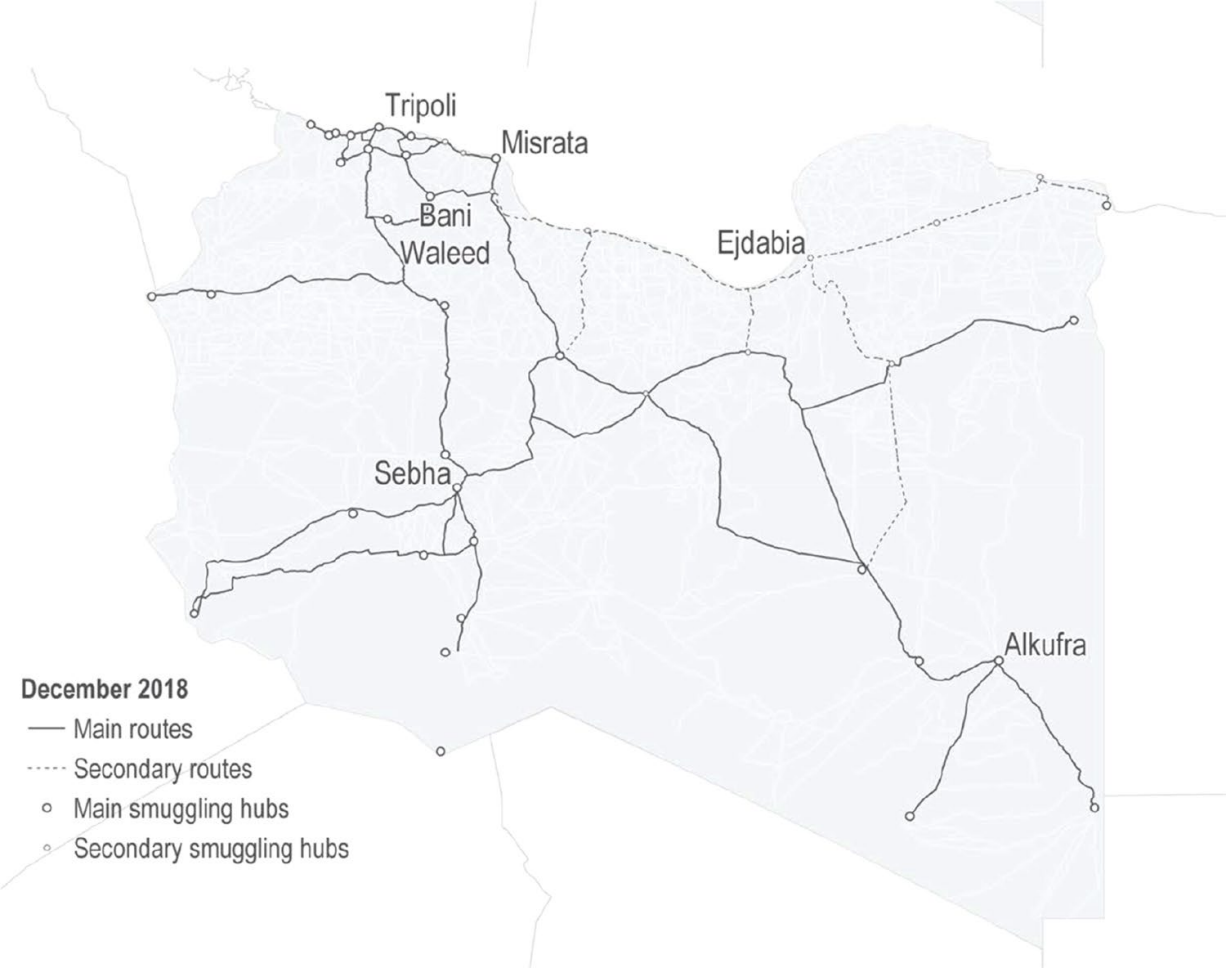
Example of migration routes from sub-Saharan Africa through Libya at the end of 2018, UNHCR, 2019 [15]

The Al Kufra district, roughly the size of Spain, is the remote Southeastern quarter of Libya. It is at the beginning of the road connecting Al Kufra City to the North. No roads extend farther to the borders of Egypt to the east, the Sudan and Chad to the South, approximately 200, 300, and 400 km away respectively, across open roadless desert, Fig. 1. The majority of migrants from different parts of Africa arriving et al. Kufra from illegal border crossings, have no required documents or work permits. Highest migrant origins include Sudan and Chad (Central Africa), the next greatest proportion of migrants entering the city originated from Burkina Faso, Niger, Mali, Nigeria, and Ghana (West Africa), and from Eretria, Somalia, and Ethiopia (Horn of Africa). Departing from the Mediterranean coast around Tripoli, ultimate destinations are the developed economies of European Union (EU) and Britain [16].

Local health officials recognize that the migrants arrive et al. Kufra in desperate condition, on foot, carrying only what has been essential for survival in crossing the desert. Infectious diseases are common including hepatitis, tuberculosis, STDs, and all manner of respiratory and enteric infections [unpublished]. In effort to control the spread of disease, migrants seeking documentation to permit work or for licenses for trade or commerce are required to obtain a health document indicating that they have presented at the local hospital for testing, the source of samples for this project. Documents must be presented at a checkpoint at the beginning of the road to the North. Despite this requirement, an unknown proportion, estimated ca. 2/3–3/4 of migrants, fearing travel restriction, do not present at the hospital and circumvent the checkpoint on foot to be picked up at a point beyond and out of sight of the checkpoint. The number of migrants trying to reach Europe from Al Kufra, estimated from hospital records and checkpoint observations, are typically 500 migrants/day, with approximately 500/week remaining in Al Kufra, 100/month returning to countries of origin, resulting in approximately 2000/week passing the checkpoint. Currently increasing, the flow of African migrants into Al Kufra city from neighboring countries, especially those arriving from intermediate and high endemic countries, is an additional burden on the local population in the host nations.

The aim of this project was to determine HBV and HCV prevalence in newly arrived African migrants arriving et al. Kufra City from sub-Saharan regions where both viral infections are highly endemic.

## Materials and Methods

This project was conducted for 12 months from January 01 to December 31, 2019, et al. Kufra City hospital laboratory in a high migrant area of Libya. Migrants arriving et al. Kufra and seeking documentation, are required to attend the local hospital for a basic health examination supervised by the Libyan National Centre for Disease Control (CDC) following national ethical guidelines. With informed consent, a 5 mL blood sample is collected to identify infectious disease conditions. At sample collection socio-demographic characteristics (age, sex, and country of origin) are recorded. Following analysis for CDC requirements, serum samples are retained briefly before disposal. The sera were provided for unlinked anonymous testing (UAT) in this project and approved by the Office of the Faculty Undersecretary for Scientific Affairs, Benghazi University-Libyan, not requiring further ethical oversight. Retained sera were stored at – 20 °C until tested. In the 2019 calendar year serum samples were analyzed from 3248 newly arrived migrants from different parts of sub-Saharan Africa.

The UAT serum samples were tested and confirmed for HBV and HCV. Screening for HBV used the “Advanced Quality™ One Step HBsAg Test” for detection of HBV surface antigen, an immunochromatographic assay for serum or plasma samples (InTec Products Inc, Xiamen, Fujian P.R. China). Screening for HCV used the “Advanced Quality™ Rapid Anti-HCV Test” for detection of anti-HCV antibodies, a colloidal gold enhanced immunochromatographic assay for human serum or plasma (InTec Products Inc, Xiamen, Fujian P.R. China). Samples that were positive for HBV and HCV further tested using the “HBsAg ELISA Kit” and the “HCV Ab ELISA Kit”, both are third-generation enzyme linked immunosorbent assays (ELISA), (BIONEOVAN Co., Ltd, Beijing, P.R. China). All screening positive samples were confirmed at least once to avoid false positive results and considered as positive only if the retest was positive. Positive and negative control serua provided with the assay kits for HBV and HCV tests were included in all assay runs following the manufacturer’s instructions to avoid false positive and negative results.

### Statistical Analysis

Data were analyzed using SPSS software version 25. Descriptive statistics were performed to describe the frequency of the different study variables, and to estimate the prevalence of HBV and HCV infections. The associations of the gender, age, and country of origin with hepatitis B surface antigen (HBsAg) and anti-hepatitis C virus (HCV) antibodies results as the dependent variable were tested using binary logistic regression. Odds ratio (OR) at 95% confidence interval (CI) was calculated and a p-value < 0.05 was considered significant.

## Results

A total of 3248 newly arrived migrants from different parts of sub-Saharan African countries were included in the study. The overwhelming majority, 96.5%, were male, only 113/3248 (3.5%) were female. Ages ranged from 18 to 53 years with a mean age of 33.1 ± 8.8 SD years. The population was divided by age into 3 groups, 18–28, 29–38, and 39–53, Table 1. The male population was roughly evenly divided between the age groups, the female migrants were predominantly in the youngest group. Few (5.3%) females tested were 39 or older, Table 1.

**Table 1:** Age and gender distribution of 3248 sub-Saharan African migrants newly arrived et al. Kufra, January to December 2019, travelling toward the Mediterranean and Europe

Population age range	Male No. (%)	Female No. (%)	Total No. (%)
39–53	1015 (32.4)	6 (5.3)	1021 (31.5)
29–38	1066 (34.0)	29 (25.7)	1095 (33.7)
18–28	1054 (33.6)	78 (69)	1132 (34.8)
Sub-Total	3135 (96.5)	113 (3.5)	3248 (100)

This migrant population was distributed in approximately equal thirds across the three age ranges. Nearly 60% of migrants in this study were from Central African countries (Chad and Sudan). Just under a third, 890 (27.5%) were from West African countries (Burkina Faso, Niger, Mali, Nigeria, and Ghana) The remainder, 457 (14%), were from the East African countries (Eritrea, Somalia and Ethiopia) (Table 2).

**Table 2:** Countries of origin of 3248 sub-Saharan African migrants newly arrived et al. Kufra, January to December 2019, travelling toward the Mediterranean and Europe

Region/Country	Total (%)	Male	Female
East Africa			
Eritrea	137 (4.2)	108	29
Somalia	199 (6.1)	174	25
Ethiopia	121 (3.7)	112	9
EA Total	457 (14)	394	63
Central Africa			
Chad	1032 (31.7)	1009	23
Sudan	869 (26.8)	848	21
CA Total	1901 (58.5)	1857	44
West Africa			
Burkina Faso	173 (5)	172	1
Niger	450 (14)	446	4
Mali	182 (5.6)	181	1
Nigeria	64 (2)	64	0
Ghana	21 (1)	21	0
WA Total	890 (27.5)	884	6
Total	3248 (100)	3135	113

Over 12-month study period the 3248 newly arrived migrants were screened for HBV and HCV. Of these, 761 were positive for HBsAg, and 1014 were positive for anti-HCV rapid tests. The same prevalence was found by the ELISA confirming test. The overall percent prevalence calculated was 23.4% HBV and 31.2% for HCV.

### HBV Prevalence

The overall prevalence of HBV in the 3248 migrant population was 23.4%. The HBV prevalence by HBsAg positivity was higher among male participants, 23.9% (750/3135) than among female participants, 9.7% (11/113), (Table 3). The difference was significant (P = 0.001) with 2.5 times as many male migrants HBV positive as female migrants (Table 3). In this population of 3248 migrants, statistically significant differences in HBV prevalence were observed by age group: 18–28 [25.35%] and 29–38 [31.6%], compared to 39 + [12.54%]. Major differences in HBV prevalence were found between regional groups, highest at 55.57% among East African migrants, 35.6% among West Africans, and 10% among Central African migrants (Table 3).

**Table 3:** Seroprevalence of hepatitis B surface antigen (HBsAg) among African migrants transiting Al Kufra, Libya, by demographic characteristics and regions of origin

Category	Prevalence of HBsAg No (%) positive negative 761 (23.4) 2487 (76.6)	OR	95% CI Lower Upper	*P* value
Age (yrs)				
39 +	128 (12.5) 893 (87.5)	1.00		
29–38	346 (31.6) 749 (68.4)	2.17	1.70 2.78	.001
18–28	287 (25.4) 845 (74.6)	1.58	1.23 2.03	.001
Gender				
Females	11 (9.7) 102 (90.3)	1.00		
Males	750 (23.9) 2385 (76.1)	3.39	1.83 6.24	.001
Region				
(EA)	254 (55.6) 203 (44.4)	10.76	8.42 13.75	.001
(CA)	190 (10.0) 1711 (90.0)	1.0		
(WA)	317 (35.6) 573 (64.4)	4.18	3.39 5.16	.001

East Africa (EA), Central Africa (CA), West Africa (WA). Confidence Interval (CI), Odds Ratio (OR), hepatitis B surface antigen (HBsAg)

### HCV Prevalence

The overall prevalence of HCV in the 3248 migrant population was 31.2% (Table 4). The prevalence of anti-HCV among male participants (30.8%) was significantly (p = 0.001) lower than female participants (41.6%) (Table 4). Statistically, significant association of HCV prevalence with migrant age group was observed: 31% among18–28 year-olds; 47.2% among 29–38 year-olds; and 14.3% among migrants 39 or older (Table 4).

**Table 4:** Seroprevalence of anti-HCV antibodies among African migrants transiting Al Kufra, Libya, by demographic characteristics and regions of origin

Category	Prevalence of anti-HCV No (%) positive negative 1014 (31.2) 2234 (68.8)	OR	95%CI Lower Upper	*P* value
Age (yrs)				
39 +	146 (14.3) 875 (85.7)	1.00	2.55 4.07	.001
29–38	517 (47.2) 578 (52.8)	3.22	1.65 2.67	.001
18–28	351 (31.0) 781 (69.0)	2.10		
Gender				
Females	47 (41.6) 66 (58.4)	1.00		
Males	967 (30.8) 2168 (69.2)	0.54	0.35 0 .84	.006
Region				
(EA)	302 (66.1) 155 (33.9)	12.06	9.46 15.38	.001
(CA)	233 (12.2) 1668 (87.7)			
(WA)	479 (53.8) 411 (46.2)	1.00 6.96	5.71 8.49	.001

*CA* Central Africa, *WA* West Africa, *EA* East Africa; *CI* Confidence Interval, *OR* Odds Ratio, hepatitis C virus (HCV)

Prevalence of HCV also varied by region of origin. Prevalence of HCV was highest among migrants East African at 66.1%, closely followed prevalence in the West African group at 53.7%. The Central African migrants showed the lowest prevalence, 12.2%. The difference in HCV prevalence between each of the geographic groups was statistically significant.

## Discussion

The HBV and HCV prevalence measured in sera of the 3248 migrants were of substantial interest compared to other reports with respect to differences in age, gender, and region of origin. In this migranst population the prevalence of both HBV and HCV was significantly lower in the oldest age group, > 39 (Tables 3 and 4). In other populations prevalence has been described as generally increasing with increasing age [17–19].

The gender-specific HBV findings (Table 3) were generally consistent with previous reports indicting higher male HBV prevalence but not to the 2.5:1 extent observed in this migrant population. The proportion of females in the population tested was very small, only 3.5% of the total. The total number of female migrants tested, 113, is large enough to provide confidence in the positive proportions reported. The high HCV prevalence observed among female migrants, 41.6%, differs from other reports indicating higher prevalence among males, and is much higher than reported in other populations [2, 17].

Significant differences in both HBV and HCV prevalence were observed between migrants grouped according to region of origin. Migrants of EA origin had highest prevalence, of 55.6 and 66.1%, also high in WA migrants, 35.6 and 55.8%, and lower in the CA population, 10.0 and 12.2% respectively. The observed levels were much higher than previously reports citing high HBV levels as > 8%, ranging to 12–15%, high HCV levels as > 2.5% ranging to “an astoundingly high” level of 14.7 in Egyptian adults [10, 20–23].

Overall, the basic features of hepatitis B and C, the diseases that they cause, the consequences of infection, and their epidemiology…distribution, prevalence by region, and risk factors…are reasonably well-established [4, 24–27]. Epidemiology of both HBV and HCV are characterized regionally by a wide range of prevalence related closely to economic, social, and sanitation conditions [28–34]. Prevalence of HBV globally ranges from < 2% (e.g., 0.2% in the USA, 1% avg in Europe) to > 8% in areas of Asia and Africa [4, 17, 18, 24, 26]. Prevalence of HCV globally ranges from < 1% (e.g., Northern Europe, Middle East, India) to > 5% (e.g., areas of Africa, central Asia, Tibet) [4, 14, 19, 27, 35]. Libya overall, where this work was conducted, has “Low Intermediate” HBV prevalence, 1.5–2%, and “Low Moderate” HCV prevalence, also 1.5–2% [20, 36]. Against the relatively low background levels of HBV and HBC prevalence in the Libyan population at large, the data presented here show that in 3248 migrants in the 2019 calendar year prevalence was much higher overall with nearly one in four (23.4%) having evidence of HBV infection and nearly one in three (31.2%) having evidence of HCV infection, but even 2 × higher prevalence in EA and WA subgroups.

Important questions in light of these data are: (1) Are the data representative of migrants? (2) Are the data representative of the population in regions of origin? (3) What is the significance for destination populations? and (4) What actions/policies do the data suggest?

1. The data are from a large population, 3248 samples analyzed over the calendar year. However, based on the estimate of arrivals et al. Kufra of ca. 500/day, the samples analyzed are a small fraction of the total population i.e., about 10/day. This represents the population presenting at the local hospital to obtain documentation, ultimately needed to pass the departure checkpoint. The population passing the checkpoint estimated at ca. 2000/month (ca. 100/day) is thus 1/4 of the total migrants passing Al Kufra. Whether the prevalence of 23% HBV and 31% HCV are representative is a matter of speculation.
2. According to published reports HBV and HCV prevalence in sub-Saharan populations varies widely depending on the specific population sampled. It appears that although both HBV and HCV rates among the general populations in countries of origin are in the range of 2–10% for HBV and 2–15% for HCV, infection rates in the migrant populations are more reflective of higher risk groups. Local Al Kufra observers describe the general condition of arriving migrants specifically et al. Kufra as depleted and sharing everything including clothing, eating, and personal care items offering ample opportunity for bloodborne exposure.
3. The significance of high HBV and HCV prevalence in migrant populations to destination country residents has been examined by European health agencies concluding that indeed, due to the high prevalence, migrant infections increase the prevalence in the overall destination countries [21]. However, due to the relative isolation of migrant groups, simple logic suggests the risk of transmission would be highest within immigrant groups and rather than to resident populations [7, 22, 23]. Overall impact is country specific related to the immigrant proportions in destination countries and to the HBV and HCV prevalence in the native populations.
4. Actions/Policies. The effectiveness of effort allocated to public health related to HBV and HCV depends on the accuracy of modelling supporting policy and resulting actions. In turn, the accuracy of modelling is controlled by input data. The ability to understand the significance of HBV and HCV introduced to destination country populations depends on understanding HBV and HCV prevalence in migrants [21]. The population studied here appears relatively unbiased, early at a collection point in migration before dispersal, and provides a unique view of the extent and magnitude of HBV and HCV infection, and potentially other infections, among migrants from the areas described.

Concentration of migrants at locations along migration routes and as collected in destination countries, informed by screening data as illustrated by this study, provides opportunity to inform those infected of consequences and risks, and to provide treatment where indicated.

This study shows significant rates of HCV and HBV prevalence in African migrants gathered in Libya from many sub-Saharan African countries, an overall estimated HBV and HCV prevalence of 23.4 and 31.2% respectively, with higher prevalence in migrants from the East African region (HCV, 66.1%, and HBV, 55.6%), and in the West African region (HCV, 53.8% and HBV, 35.6%) and lower rates in the central African region (HCV, 12.3% and HBV, 10.0%). The regional hepatitis B surface antigen (HBsAg) and HCV-antibody estimates in our study were higher than those reported in many sub-Saharan countries of Africa. This suggests that the migrants included in our study, ones sufficiently desperate to risk the journey, have differed from the populations at large in their countries of origin. Including broader infection screening in conjunction with required migrant screening would permit individual attention indicated by test results.

## Limitations

The population sampled was limited to those migrants complying with the requirement for those seeking documentation to present at the local clinic for health screening. Those complying are estimated to be on the order of 10–20% of migrants passing through the Al Kufra region and not necessarily representative of HBV and HCV infection rates in all migrants.

The number of females in the population tested was less than 5% of the total. Although an accurate count of migrants passing through the Al Kufra region is not maintained, the proportion of migrant females at this location is typically a small minority.

Testing for HCV infection was not confirmed by RNA analysis and therefore may be an over estimate of those having active communicable infections.

Due to the nature of the UAT sample access for HBV and HCV analysis, follow up with infected individuals was not possible.

## Conclusion

Migrants originating in sub-Saharan Africa are of great interest because the region is reported to have the high HBV and HCV prevalence rate. This study shows clearly that HBV and HCV prevalence in migrants significantly exceed even the relatively high viral hepatitis prevalence sub-Saharan African countries. We measured the overall HBV prevalence of 23.4% and HCV prevalence of 31.2% in a large cross-section of the migrant population. The findings of this study suggest that migrants from high HBV and HCV prevalence countries represent an important risk group for HCV and HBV infection as they proceed toward and integrate into European destination populations.
